# Seven computations of the social brain

**DOI:** 10.1093/scan/nsab024

**Published:** 2021-02-25

**Authors:** Tanaz Molapour, Cindy C Hagan, Brian Silston, Haiyan Wu, Maxwell Ramstead, Karl Friston, Dean Mobbs

**Affiliations:** Department of Humanities and Social Sciences, California Institute of Technology, Pasadena, CA 91125, USA; Department of Humanities and Social Sciences, California Institute of Technology, Pasadena, CA 91125, USA; Department of Psychology, Columbia University, New York, NY 10027, USA; Department of Humanities and Social Sciences, California Institute of Technology, Pasadena, CA 91125, USA; CAS Key Laboratory of Behavioral Science, Department of Psychology, University of Chinese Academy of Sciences, Beijing, 10010, China; Department of Psychology, University of Chinese Academy of Sciences, Beijing, 10010 China; Division of Social and Transcultural Psychiatry, Department of Psychiatry, McGill University, Montreal, Quebec H3A 1A2, Canada; Culture, Mind, and Brain Program, McGill University, Montreal, Quebec H3A 1A2, Canada; Wellcome Centre for Human Neuroimaging, Institute of Neurology, University College London, London WC1N 3AR, UK; Wellcome Centre for Human Neuroimaging, Institute of Neurology, University College London, London WC1N 3AR, UK; Department of Humanities and Social Sciences, California Institute of Technology, Pasadena, CA 91125, USA; Computation and Neural Systems Program, California Institute of Technology, Pasadena, CA 91125, USA

**Keywords:** mentalizing, social signaling, active inference, external/internal self

## Abstract

The social environment presents the human brain with the most complex information processing demands. The computations that the brain must perform occur in parallel, combine social and nonsocial cues, produce verbal and nonverbal signals and involve multiple cognitive systems, including memory, attention, emotion and learning. This occurs dynamically and at timescales ranging from milliseconds to years. Here, we propose that during social interactions, seven core operations interact to underwrite coherent social functioning; these operations accumulate evidence efficiently—from multiple modalities—when inferring what to do next. We deconstruct the social brain and outline the key components entailed for successful human–social interaction. These include (i) social perception; (ii) social inferences, such as mentalizing; (iii) social learning; (iv) social signaling through verbal and nonverbal cues; (v) social drives (e.g. how to increase one’s status); (vi) determining the social identity of agents, including oneself and (vii) minimizing uncertainty within the current social context by integrating sensory signals and inferences. We argue that while it is important to examine these distinct aspects of social inference, to understand the true nature of the human social brain, we must also explain how the brain integrates information from the social world.

## Introduction

At ∼300 000 years of age, the human brain is relatively young. Yet, its mid-Paleolithic introduction was preceded by millions of years of evolution. Through this process, over phylogenetic timescales, the brain has slowly acquired models of an increasingly complex social world, the accumulation of which has resulted in the human brain we possess today. The evolution of the human brain evolves the exploitation of group-living strategies, which benefit both the individual and the group (Silston *et al.*, 2018). In short, humans have evolved a set of behavioral and neural systems that facilitate group living and successful social interaction. These systems must be sufficiently flexible to navigate the fleeting social environment (Lehmann *et al.*, 2007), to track the behaviors, interactions and intentions of others, and to accumulate this information over time to inform and make appropriate social decisions. To understand the recruitment of specific neural systems and predict the behaviors of others, we must also account for contextual factors and sociocultural dynamics. To enable adaptive forms of social interaction, the human brain must be dynamic, efficient and attentive to—and capable of—the deployment of appropriate social behaviors in a variety of social contexts.

Like all nervous systems, the human brain has evolved primarily for survival, i.e. its main function is the guidance of situationally appropriate forms of action, which maintain it in the neighborhood of states that characterize the human phenotype (Cisek, 1999; Mobbs *et al.*, 2015; Badcock *et al.*, 2019). However, the relative size, ability and metabolic demand of the brain—and its unique capacity for language and mentalizing—suggest that the selection pressures, to which humans are subject, relate primarily to the constraints on group living. Indeed, a large portion of the human brain is dedicated to social cognition. For example, brain imaging and neuropsychological studies of individuals with brain damage suggest that the extrastriate cortices—including the visual fusiform cortex—comprise regions that specialize in the processing of faces and bodies (Kanwisher and Yovel, 2006). Social attention and the dynamic features of the human face (e.g. expression and emotion) are key elements of social interaction and encompass the superior temporal sulcus (STS; Haxby *et al.*, 2002; Hagan *et al.*, 2009, 2013).

As one ascends cortical hierarchies, computational processes become more distributed and complex. Inferences concerning the mental state and intentions of others appear to engage the temporoparietal junction (TPJ), the temporal pole as well as the medial prefrontal cortex (PFC). The emotional states of others map onto one’s own affective and interoceptive circuitry (Singer *et al.*, 2004). The perception of threat to another engages the amygdala (Adolphs *et al.*, 1995), while the perception of another’s joy engages the reward circuitry (Mobbs *et al.*, 2009). Social motivation is an important driver of social actions; however, information processing pathways have been found to differ between individuals from different cultures, underscoring the complexity, sociocultural variability and plasticity of the organ enabling human social cognition (Han and Northoff, 2008). This brief introduction to the social brain suggests that social behavior involves a diverse yet interconnected network (i.e. a heteroarchy) in the human brain and involves several specialized hubs, each with its own specialization, and each working in concert to accomplish global computations (Anderson, 2014).

In this paper, we outline seven key computations with which the social brain contends in social interaction. These include (i) social perception, (ii) social inferences, (iii) social learning, (iv) social signaling, (v) social drives, (vi) social identity and group membership and, finally, (vii) integrating interoceptive, exteroceptive and proprioceptive signals within the social context. These challenges suggest that social behavior is a cognitively complex and metabolically demanding process, which involves highly interconnected systems that pass messages over both short- and long-range connections (i.e. intrinsic and extrinsic connectivity, respectively). We argue that while it is important to examine these different computations, in order to better understand the true nature of the human social brain, we must first understand how the brain integrates multimodal information, and in turn, how this integration underwrites the enormous variety of social behaviors.

### Social perceptual systems

The human sensory system, as all other sensory systems, views the external world through the lens of evolved adaptions (Haselton *et al.*, 2015). Some have argued that identity is crucial to social interaction and that, therefore, it is not surprising that a specialized system has evolved to perceive social signals, such as facial expression, body stance, language, tone of voice and chemosensory signals (Haselton *et al.*, 2015). Research from cognitive neuropsychology—as well as human brain imaging—has demonstrated that the brain has specialized systems that process information about faces, bodies, odors and biological sounds and movements and that the human body has coevolved along with these cognitive adaptations (Kanwisher and Yovel, 2006; de Gelder *et al.*, 2010). This is borne out by a host of adaptations (both morphological and cognitive) that, in humans, are hard wired. For instance, it has been shown that newborns have the propensity to attend to faces and determine the chemosensory signals of the mother (Johnson *et al.*, 1991). Even as infants, humans have a propensity to track the gaze of their conspecifics (Batki *et al.*, 2000); this is a cognitive adaptation that coevolved in humans with a complementary phenotypic trait, namely, our highly visible white sclera (Henrich, 2016). Neuroimaging studies have shown that we engage distinct neural circuits when distinguishing between those who are similar and dissimilar to ourselves (Mitchell *et al.*, 2006; Mobbs *et al.*, 2009; Sui *et al.*, 2013; Lockwood *et al.*, 2018), determine social status, infer who to cooperate with and even whom to dehumanize (Harris and Fiske, 2006). To survive, people need an accurate perceptual system to infer states of affairs in a social and cultural econiche (Table 1).

**Table 1.** Examples of how the human perceptual system has evolved to decipher perceptual cues across diverse social landscapes.

**Detecting social danger.** Humans are particularly attentive to social expressions of threat, whether by direct expression of anger or indirectly via the observation of fear in others (Calder *et al.*, 2011). Although humans are only minimally affected by predatory attacks from other animals, our predatory defense systems have evolved to cope with social threats arising from members of our own species. In our social environment, an angry face—or antagonistic tone of voice—presents robust cues that others are aggressive and possibly dangerous (Ceravolo *et al.*, 2016).
**Detecting kin and group members.** The detection of kinship and of conspecifics is crucial for survival in humans. Evolutionary models show that people favor behaviors that benefit others who share genes. Kin detection is certainly observed in more basal species and increases exponentially in complexity as one moves to more socially complex creatures. Dawkins (1989) proposed the ‘green beard effect’, suggesting that animals, and potentially humans, possess recognition alleles that aid in the visual detection of genetically similar individuals.
**Detecting disease and health.** Especially before the invention of modern antibiotics, it was critical to avoid highly infectious diseases, such as ebola, smallpox and influenza (i.e. contamination fears). According to the disease-avoidance model, disgust functions to protect us from contiguous diseases (Oaten *et al.*, 2009). Studies indicate that people can detect disease from both physical cues (e.g. others’ appearances and behaviors) and psychological cues (e.g. ‘depressed’ *vs* ‘not depressed’). Facial (e.g. facial masculinity and maturity), vocal (e.g. pitch and tone of voice) and body (e.g. motion and movement and speed) features can signal physical strength/weakness (Fink *et al.*, 2007; Sundelin *et al.*, 2015; Von Kriegstein *et al.*, 2006).
**Fitness and beauty.** Most females and males want to copulate with those that exude beauty and health, which is a proxy for ‘good genes’ (Buss, 2016). Facial attractiveness is a facial attribute that conveys significant biological advantages (Shen *et al.*, 2016) [e.g. as expressed in mating success (Pashos and Niemitz, 2003), earning potential (Frieze *et al.*, 1991) and longevity (Henderson and Anglin, 2003)]. There is a long line of research showing that the waist–hip ratio is a predictive measure of female attractiveness (Singh, 1993), while height, body shape and penis size in males predict female attraction (Mautz *et al.*, 2013).
**Trust and cheaters.** The ability to spot cheats, free-riders and the complementary capacity to trust others and evaluate the grounds for such trust is crucial for mutualism. Several studies have shown that some faces are perceived as more trustworthy than others (Winston *et al.*, 2013). Stirrat and Perrett (2010) showed that men with greater facial width were more likely to exploit the trust of others. This suggests that facial phenotypes provide good indicators of another’s trustworthiness. Rhodes *et al.* (2013) found that women are better at predicting unfaithfulness than men and that perceived masculinity was the most dominant cue in detecting cheaters. Barkow,Cosmides and Tooby (1992, 2004) have proposed the existence of a cheater-detection module, and this has been supported by research showing that people have enhanced memory for cheaters (Bell and Buchner, 2009); similar proposals include a module for evaluating the trustworthiness of others, a so-called suspicion system (Gold and Gold, 2015).
**Protection and competence.** Todorov *et al.* (2005) showed that ratings of a political candidate’s face predicted electoral success. Others have shown that ratings of leadership ability from CEO faces predicted company profits (Rule and Ambady, 2008). It has been demonstrated that ratings of perceived competence of others (i.e., their ability to protect us) in a potentially threatening situation is a crucial component of threat assessment, which can influence levels of anxiety and defensive actions. For example, functional MRI studies show that under threat of pain, neural systems involved in pain anticipation show reduced activity when subjects rate others as higher in competence (Tedeschi *et al.*, 2015). This suggests that inferences of competence act as predictors of protection and reduce the expectation of physical harm.
**Status and dominance.** Alan Fiske has proposed that during social interactions, individuals rank authority by ‘attending to their linear order’. Nonhuman primates will pay to view social images of high-status individuals (Deaner *et al.*, 2005). Our own work has indicated that people show more conformity to individuals with higher reputations—manipulated by reputation ratings in uncertainty decisions (Qi *et al.*, 2018).

While the existence of functionally specialized systems that allow us to account for these remarkable perceptual abilities remains contentious, it is clear that there is overlap in the neural circuits involved in inferring information from faces. The face processing system is often portrayed as a hierarchically organized system. In this system, the STS, the occipital face area (OFA) and the fusiform face area (FFA) have been found to be a part of the so-called core network for face perception (Haxby *et al.*, 2000; Fox *et al.*, 2009; Kadosh *et al.*, 2011). The link between the OFA and FFA has been associated with processing facial identity, whereas the link between OFA-STS has been associated with processing the dynamic aspects of the face that contribute to recognition (e.g., expression) (Gobbini and Haxby, 2007; Olivares et al., 2015). The STS has been proposed as a hub, comparator and integration center for a host of functions, which situates it as a major contributor to social processing and behaviors (Hagan *et al.*, 2009, 2013). More fine-grained investigation by Deen *et al.* (2015) and Lahnakoski *et al.* (2012) suggests that anterior and posterior parts of the STS are nodes in different circuits subserving specific components of social information processing, with some subareas participating in multiple circuits corresponding to different categories of social input. These authors characterize the anterior region of the STS as part of a circuit involved in processing communicative signals and the posterior region as a social processing control node that is connected with areas implicated in attentional control. This structure is important for action understanding but is not necessarily activated in non-action-oriented mentalizing (i.e. false belief tasks) (Gobbini *et al.*, 2007). In addition to these ‘core’ areas, the extended systems [limbic areas, auditory regions and regions involved when processing theory of mind (ToM)] work together with the ‘core’ system to provide more complete face-driven processing, which includes the processing of social information (Haxby *et al.*, 2000). Growing evidence suggests an important role for the anterior inferior temporal (aIT) lobe in face processing, which appears to support facial recognition (Kriegeskorte *et al.*, 2007; Rajimehr *et al.*, 2009; Nestor *et al.*, 2011; Pyles *et al.*, 2013). The crucial role of the aIT in face recognition has been further supported by a study involving individuals with congenital prosopagnosia. This study showed a significant reduction in the volume of white/gray matter in the anterior IT cortex, which was correlated with deficits in face recognition (Behrmann *et al.*, 2007).

### Social inferential systems

Individuals use social perceptual systems to form general impressions of others; however, people can use mentalizing skills to make social inferences. A key process in successful social interactions is integrating body language cues, verbal information and context to furnish insight into another’s mind. Tamir and Thornton’s 3D model which suggests a three-layer structure in which the first layer describes others’ observable actions and the second and third layers concern their mental states and traits, respectively (Tamir and Mitchell, 2012). They propose that the probabilistic trajectories within, and between, these layers offer an explanation for how people might use their social knowledge to predict others’ futures.

Adjacent to the 3D is the interactive mentalizing theory (IMT), which proposes that during dynamic social interaction, four key processes are in play: (i) meta-cognition: confidence about one’s mentalizing ability (e.g. how confident Agent A is about their inference of another’s thoughts and intentions; (ii) first-order mentalizing: mentalizing of another’s mental states (e.g. what Agent A thinks Agent B’s thoughts and intentions are), (iii) personal second-order mentalizing: mentalizing of self-generated mental states from the perspective of others (e.g. how much insight Agent A thinks Agent B has into his/her own thoughts and intentions) and (iv) collective mentalizing, where we conform to what we believe another agent thinks about Agent B (e.g. Agent A infers that Agent C thinks that agent B has bad intentions) (Wu *et al.*, 2019). The latter aspect has been developed under the rubric of ‘thinking through other minds’ (Veissière *et al.*, 2019). The IMT model proposes that people are prone to this type of bias; especially, when their confidence (metacognition) is low (Qi *et al.*, 2018). During real-time social interactions, these four mentalizing components interact to update beliefs about another’s intentions (Wu *et al.*, 2019). The IMT, therefore, suggests that multiple computations are involved in social inferences (i.e. integration of social information).

These theories support a network that encodes social knowledge, which includes thinking about mental states, making inferences about others’ beliefs, thinking about the context including groups of people (Mitchell *et al.*, 2006; Ames *et al.*, 2008; Saxe and Kanwisher, 2013). This network includes dorsomedial prefrontal cortex (dMPFC), ventromedial prefrontal cortex (vmPFC), medial parietal cortex, TPJ and the anterior temporal lobes (ATLs). The medial PFC is a brain area involved in mentalizing but has also been implicated in person perception, action monitoring, expectations and metacognition (Amodio and Frith, 2006). The temporal poles and TPJ are also components of the mentalizing circuit. Activity in the TPJ has been associated with inferring the mental states of others (from one’s own perspective) but is also associated with cues indicating agency more generally (Wurm and Schubotz, 2018). For example, Saxe and Kanwisher (2013) found that descriptions of mental states recruited the TPJ, but physical descriptions of people did not—and Castelli *et al.* (2000) found TPJ activation in a task in which moving shapes appeared to possess intentionality but not for simple goal-directed actions or randomly moving shapes. While the involvement of STS and TPJ is supported by neuroimaging and brain lesion work (Samson *et al.*, 2004; Saxe *et al.*, 2004), the exact role of these brain regions is still unclear. It is possible that STS is involved in action observation and understanding and TPJ is involved in inferring different mental states (e.g. effort during action observation). Other brain regions thought to comprise the ToM network that include the precuneus and posterior cingulate, which are associated with self-referential thoughts and cognitions, such as feelings of causation or attribution to oneself (Cabanis *et al.*, 2013). Much like STS, the anterior and posterior parts of the precuneus appear to underwrite different processes in social inference. For example, in an attributional bias task, the posterior precuneus is associated with self-reference in general, while self-attributed positive *vs* negative sentences elicited activation of the anterior part of the precuneus (Cabanis *et al.*, 2013). The precuneus is also involved in updating state self-esteem by transforming others’ evaluation of oneself into state self-esteem, thereby relating to the mentalizing system for subjective evaluation regarding others (Kawamichi *et al.*, 2018).

### Social learning systems

The philosopher Gilbert Ryle proposed that a boy can learn chess by simply ‘watching the moves made by others’ (The Mind: p41). Social learning is a major benefit when living in groups, accelerating overall learning and leading to adaptive solutions that can be passed on to offspring and other conspecifics over developmental timescales. Animals that cannot imitate others are confined to the rules of individual learning (Richerson and Henrich, 2012). Whiten (2005) suggests that social learning provides a ‘secondary inheritance system’, where our capacity to learn from others lowers the cost of acquiring information firsthand, including learning about dangers, cheaters and the best locations to forage [a complementary account from the perspective of human evolutionary biology is provided by Henrich (2016)]. Therefore, specialized brain systems seem to exist that support the computations involved in social learning. Below we will outline selected findings on how the brain signals self- and other-referenced social learning.

#### The Anterior Cingulate Cortex.

The anterior cingulate cortex (ACC) has been proposed to be an integrative area relating to social learning systems (Lockwood *et al.*, 2020). Specifically, the ACC seems to be involved during social decision-making, reflecting information processing about self, other, or both (Apps and Ramnani, 2014; Lockwood *et al.*, 2015; Apps *et al.*, 2016; Hill *et al.*, 2016). In one recent study, the whole ACC was lesioned in rhesus monkeys where they found specific disruption of learning which stimuli rewarded others, but not the self, while previously learned stimuli were still intact (Basile *et al.*, 2020). These findings indicate the importance of the ACC when acquiring prosocial preferences from vicarious reinforcement. Moreover, neuroimaging studies in humans suggest an important division between social and nonsocial subregions within the ACC, namely the sulcus (ACCs) and gyrus (ACCg) (Chang *et al.*, 2013; Apps *et al.*, 2016; Joiner *et al.*, 2017; Kendal *et al.*, 2018). Several studies have found that the ACCg plays an important role in evaluating the behaviors of others, estimating other’s level of motivation and error processing, whereas the ACCs responds to self-relevant reward signals and prediction errors (Apps *et al.*, 2016; Chang and Sanfey, 2013; Hill *et al.*, 2016; Lockwood *et al.*, 2016). Learning about reward probability from vicarious and personal experiences does seemingly recruit other neural systems where the information gets combined when making decisions.

#### The Ventromedial Prefrontal Cortex.

The vmPFC is also implicated in vicarious reward learning (Mobbs *et al.*, 2009), vicarious prediction errors (Burke *et al.*, 2010) and vicarious fear learning (Olsson *et al.*, 2007; Olsson and Phelps, 2007). These studies point to the PFC as another crucial player in social learning. Although the exact processes are unknown, Price and Boutilier (2003) have put forward a Bayesian imitation model of the PFC, stating that humans (and possibly other animals) combine the information learned through the observation of others with existing knowledge afforded by personal experiences (also see Dunne and O’Doherty, 2013) and behave accordingly. The development of vicarious learning systems has roots in representational processes that recruit motor, affective, sensory and cognitive systems associated with first person experiences while observing others performing actions, perceiving sensations or under distress. The so-called mirror neuron system purports to provide a vicarious experience to observers, though the interpretation of exactly what this system does is still under debate (Cook *et al.*, 2014). While it is clear that these observations are represented in some regard to areas that are active when we perform similar actions, how this information is integrated into action understanding is not well understood. Nonetheless, the recognition of various actions of others, together with an explicit representation of their goals and our own knowledge, seems sufficient to generate a framework for vicarious learning (for an extensive review, see Charpentier and O’Doherty, 2018; Konovalov *et al.*, 2018).

### Social signaling systems

Thorndike (1920) proposed that social intelligence rests on two central properties: the ability to understand others and the behavioral effectiveness of social actions. Social signals are driven by the importance of conveying information and are observed with varying complexity across the animal kingdom (Dawkins and Krebs, 1978). Social signals are conveyed via multimodal cues such as intonation, posture, intensity, gaze direction, etc., and reduce the asymmetry of information between the signaler and receiver. However, they can also be used by the signaler strategically to promote a desired image for personal status-seeking. Signaling theory has been used to explain behavior in several fields including economics (Spence, 1973) as part of game theory, anthropology—with respect to selecting costly behaviors that otherwise appear irrational—and biology, as an evolutionarily adaptive strategy to gain or communicate social status and mitigate potential harm (Zahavi, 1975, 1977; Grafen, 1990). Subconscious signals are expressed through body language, facial expressions, touch or tone of voice. These signals include brain areas involved in language, motor and control systems.

#### Inner self: self-monitoring, metacognition and control.

Lieberman (2007) suggests that one central question for social neuroscientists is ‘how do we control ourselves’?. Baumeister and Vohs (2004) propose that humans have an innate capacity to regulate and alter their social behavior in reference to external guidelines. These guidelines include social norms, religion, morals, contextual rules and the law. Some have even gone so far as to propose that humans always are thinking in terms of expectations, and especially, what others expect of us and what are our personal expectations (Veissière *et al.*, 2019). One important part of this internal process is metacognition, or the knowledge that we have about our internal cognitive processes, which plays a key role in the control and monitoring of the internal-self (see Metcalfe and Shimamura (1994) for detailed review). Successful self-monitoring and control require coordinated activity in prefrontal circuits to override the connection between the value signal and motivation systems that lead to action selection.

The inability to control and monitor one’s behavior is typically impaired in patients with prefrontal damage (Damasio, 1995) and susceptible to failure upon depletion of self-regulatory resources. Regulatory failure has also been associated with reduced dorsolateral prefrontal cortex (DLPFC) activity and with functional connectivity between the inferior frontal gyrus (a region implicated in certain elements of response inhibition) and the vmPFC and orbitofrontal areas (regions thought to encode the value of reward) (Dambacher *et al.*, 2015; Stramaccia *et al.*, 2015). Retrieval of meta-goals—or those associated with personal longer-term outlooks, and unaccomplished by any single decision or action—may be central in influencing self-regulatory behaviors. Lateral frontopolar regions are implicated in high-level cognitive monitoring and representation in the tracking of meta-goals, with medial subdivisions involved in memory processes that are likely required to retrieve particular goal information (Baird *et al.*, 2013). Together, these internal processes will determine the behavioral output or the external presentation of the self.

#### External presentation of self: speech and nonverbal signals.

Inferences about the internal self set the foundation for social interaction. The use of language in social interaction is beyond the scope of this review; however, several core features deserve a special mention. Bolinger (1965) spoke of speech metaphorically as an ocean, where forces acting on it create surface movements, resembling the ups and downs of the human voice. Like the ocean, speech conveys voluminous undercurrents, including assertiveness and confidence through rising pitch; it transmits emotion through prosodic tone, status through grammatical accuracy and dialect, and intelligence through vocabulary and pronunciation. Therefore, what we say and how we say it are rich sources of social information. This weaving of transient social information is augmented by visual information that includes the infinitesimal movements that characterize the complex facial muscles, movement and directionality of the eyes, gait, hand gestures, speed of movement, proxemics and so forth. Humans are acutely aware of how we are viewed by others, and in many cultures, individuals accumulate and display fine material belongings to signal wealth, which is a proxy for high social status. Bourdieu famously argued that material signaling consisting of one’s ‘symbolic capital’ could be used interchangeably with economic capital to acquire social status, including advantageous positions vis-à-vis access to high-quality mates, ability to forge advantageous and stable alliances and enhanced opportunity to acquire additional status (Bourdieu, 1977).

While we elicit all these signals, the human brain is encoding others’ social signals, inferring allowable subsequent behaviors based on these signals and prior knowledge and is making social judgments concerning the target individual’s intentions. For example, Keltner *et al.* (2014) have shown that humans exhibit nonverbal signs of a prosocial character. These signals include smiles head nods, head tilts, blushing and laughter that collectively may indicate social engagement, warmth and concern for others (Keltner *et al.*, 2014). Another social cue is proximity, which provides information about the connectedness of people, where close others (or those we selectively bond with) place themselves (and are allowed to place themselves) within our personal or intimate space (Hall, 1966).

#### Consistency in representations of the inner and external self.

Festinger and Carlsmith (1959) defined internalization as the process of matching one’s private self-concept with one’s external behavior. Several theories have been advanced to account for the relationship between internal and external selves. Self-verification suggests that people act in ways that are consistent with how they self-identify (Swann *et al.*, 1987). This is closely allied with self-discrepancy theory (SDT; Higgins, 1987). SDT proposes that individuals have an internal self-model, to which they compare their behavior. Self-guides include the actual self, ideal self and ought self. SDT further predicts that when self-guides are incongruent, emotional discomfort will emerge. Therefore, one goal during social interaction is to minimize the discrepancy between internal and external states. This is evident when one feels a mismatch between goals and their attainment (e.g. rejection). The systems underlying this feeling may share common neural substrates with dissonance, more generally, which is assumed to provide an uncomfortable feeling that motivates our actions and desire to return to a coherent state. Cognitive dissonance, according to (Festinger, 1962), recruits areas involved in error conflict monitoring, notably the ACC, but also regions associated with affect and memory processing, including the insula and precuneus (Kitayama *et al.*, 2013; De Vries *et al.*, 2015).

#### Shared reality: rapport forming and social tuning.

Shared reality theory posits that when we take another person’s perspective, we become socially attuned and possess a mutual understanding (Hardin and Higgins, 1996). Rapport is critical to cooperation and conflict resolution and can be considered a form of social bonding (see above). Forming a stable rapport typically increases the overlap of beliefs and emotional responses between individuals—leading to an intrinsically rewarding interaction, providing an incentive to expend significant energy to maintain a positive shared experience. This shared reality results in affiliative behaviors, social bonding and shared epistemic needs (Hardin and Higgins, 1996) and is crucial for healthy social and psychological functioning (Echterhoff *et al.*, 2009). A salient feature of the promotion system is affiliative motivation (Sinclair *et al.*, 2005). Socially tuned interactions should produce characteristic social behaviors, including behavioral mirroring. However, social anti-tuning, as engaged by the prevention system, should be evidenced when people aim to distance themselves from others, as occurs with out-group or individuals who perceive themselves to be of lower status than others (Sinclair *et al.*, 2005).

### Social motivation system

From amoeba to humans, rewarding states are approached and pain is avoided (Higgins, 1997). Extending this dichotomy social behavior, regulatory focus theory (RFT) suggests an individual’s motivation interacts with goal pursuit (Higgins, 1987). RFT parses motivation into either a promotion focus, where one focuses on nurturance needs and gain *vs* non-gain situations, or a prevention focus, which emphasizes security needs and non-loss *vs* loss situations. Therefore, the promotion–prevention system would be engaged when one attempts to optimize social drives, through bonding, social tuning and biasing, and social network formation. For example, when status-seeking is in progress, promotion would presumably result in socializing with high-status individuals and prevention through avoiding or limiting interactions with low-status individuals. Assimilation and allegiance are also important promotion motivators. These drives would presumably be enabled by the well-known circuitry involved in motivation, including the dopaminergic and opioid circuitry in the basal ganglia and ventral tegmental area (Berridge and Robinson, 1998). Further, some theorists have suggested that the left hemisphere is associated with affiliative and promotion-type behaviors and parasympathetic activation, while the right hemisphere produces aggressive, defensive and prevention-type behaviors and sympathetic activation (Craig, 2005). Craig’s model derives from two premises: the fact that autonomic projections to the heart are asymmetric; and the idea that the brain, given its high metabolic consumption rate, requires optimization of energy consumption to perform at its observed level. This model highlights a key role for the insula, given its position as a hub and connections with areas subserving opposing components of the autonomic nervous system.

#### Social promotion and reward.

In humans, social rewards tap into the same dopaminergic systems involved in primary rewards such as food and sex (Izuma *et al.*, 2008). Indeed, the drive to broadcast information about themselves, (Tamir and Mitchell, 2012), to be liked (Davey *et al.*, 2010) and to have a positive reputation (Izuma *et al.*, 2008) increase activity in the dopamine-enriched ventral striatum (VS). In addition to the VS, the vmPFC has also been widely implicated in social reward and play an important role in value-based learning and decision-making in general (Bartra *et al.*, 2013). Advice giving may be one way in which individuals can gain the most basic of social rewards: acceptance and respect (Baumeister and Leary, 1995). This was investigated by examining advice acceptance and reflected glory (Mobbs *et al.*, 2015). In this study, it was shown that activity increased in VS when one’s advice was accepted in a three-player advisor–advisee game. Furthermore, if this advice led to the advisee winning money, activity in the VS also increased, suggesting that it is rewarding to see others win if it reflects positively on our advice (Mobbs *et al.*, 2015). Therefore, the human propensity to provide others with advice may act as a positive, status-enhancing behavior. Another study directly investigating reward-related neural activity in monetary and social rewards found common activation in VS during reward anticipation, but divergent results during reward presentation, with monetary and social rewards associated with greater thalamic and amygdala activity, respectively (Rademacher *et al.*, 2010).

#### Social prevention and punishment.

The most commonly studied form of social punishment is that of ostracism. Social pain and rejection motivate people to avoid exclusions and conform with others (Lin *et al.*, 2018), which involves the same neural networks (e.g. VS and vmPFC) as when tracking reward signals, updating value information and motivating people to act (Klucharev *et al.*, 2009; Zaki *et al.*, 2011; Nook and Zaki, 2015). In a set of classic studies, Eisenberger, Lieberman and Williams have shown that when subjects are ignored by other players in a three-player cyberball catch game, they report feeling social pain (Eisenberger *et al.*, 2003). This feeling of social rejection correlates with increased neural activity in brain regions known to be involved in physical pain (Eisenberger *et al.*, 2003). Other studies investigating social exclusion have identified the lateral and medial prefrontal cortex (mPFC), several subregions of the ACC and insula (Gunther Moor *et al.*, 2012). Similar regions have been found to activate when people feel envy (Takahashi *et al.*, 2009) and guilt (Chang *et al.*, 2011). Social punishment and forgiveness of excluders has been shown to activate regions implicated in mentalizing and ToM, (Will *et al.*, 2015) including the TPJ, STS and several areas of the PFC, and the pre-supplementary motor area. This is likely because it entails taking the perspective of and making inferences about others’ mental states, both of which are critical for empathy and cooperation (Heatherton, 2011). In third-party determination of appropriate punishments for crimes committed, some have found activity in the amygdala, mPFC and posterior cingulate cortex (PCC) when subjects assessed magnitude, and activity in the right dlPFC when determining culpability (Sebastian *et al.*, 2011). The social pain network may work to drive the reward network via retaliation or revenge.

#### Affiliation and social bonding systems.

In humans, significant mother–infant interaction is associated with synchrony in various biological rhythms such as heartbeat (Feldman *et al.*, 2011) and other autonomic coupling that reflects a shared affective state (Ebisch *et al.*, 2012), although these may be influenced by attachment security (Waters and Mendes, 2016). More recently, Preston (2013) has pointed out that mammals are attuned to, and motivated to help, neonates when they produce signals of distress. As mentioned above, this drive may be higher in females, as stress increases tending behaviors (Taylor *et al.*, 2000). The biological mechanisms that underlie the tend–befriend systems are grounded in the attachment–caregiving system, which is involved in maternal bonding and rearing. Oxytocin is believed to be the core biological chemical that facilitates mother–infant attachment (Drago *et al.*, 1986; Preston, 2013). In human mothers, viewing their own infant’s faces during fMRI scanning resulted in activation of oxytocin-enriched regions of hypothalamus and pituitary gland (Strathearn *et al.*, 2009). Others have shown that images of increasingly cute baby faces result in increased activity in dopaminergic rewards areas, suggesting that these images provide an innate primary reward (Glocker *et al.*, 2009). Consistent with this model, the insula is modulated by oxytocin signaling (Riem *et al.*, 2011) such that increased signaling upregulates insular activity and downregulates amygdala activity. Most bonding research involves mother–infant dyads; however, some studies point to gender differences or lack thereof in the affective and motivational systems that drive parental bonding behaviors (Rajhans *et al.*, 2019).

### Group identity and bias

People quickly evaluate and use social categories (e.g. race, gender, status and age)—not always based on perceptual features as discussed in the section social perceptual systems above—as a guide on how to interact with others (Ramstead *et al.*, 2016). Social groups give individuals a sense of social identity, which is based on the group to which they belong—and is a strong determinant of how one reacts to the observed outcomes of others. Others perceived as similar to oneself, and therefore as belonging to the same social category, generate both behavioral and neural increases in vicarious reward processing, even when others are not genetically related (Mobbs *et al.*, 2009). Perception of self-similar others activates neural regions including the ventral mPFC, which is also recruited during self-referential thought, while more dorsal areas of the mPFC are associated with perception of dissimilar others (Mitchell *et al.*, 2006; Sul *et al.*, 2015; Wittmann *et al.*, 2018; Piva *et al.*, 2019). Social orientation toward others and ensuing behaviors may be determined in part by the location of mPFC activation during perception of others. Specific mPFC location may bifurcate the simulation processing to proceed under the assumption the other is ‘like me’ or ‘not like me’ (assuming no other inputs). However, activation location can be shifted toward self-referential representation as a result of perspective taking of others that may have initially been perceived as dissimilar to oneself (Ames *et al.*, 2008; Nicolle *et al.*, 2012). This and other evidence suggest that social group categorizations can be quite flexible in general. This has also been demonstrated with minimal group paradigms, where individuals are randomly assigned to previously unfamiliar social groups based on arbitrary cues (e.g. a color) associated with a group. The surprising results indicate how easily biases in favor of arbitrary in-groups occur (Otten, 2016). However, it should be noted that evaluative preferences with respect to real groups tend to be stronger than those observed with minimal groups (Dunham, 2018).

Individual responses to socially relevant information can be biased depending on from whom the information is coming (i.e. ingroup *vs* outgroup). For example, participants who identified as strong supporters of a political party rated identical statements as more inspirational if they believed the statements originated from their ingroup (*vs* outgroup) leaders (Molenberghs and Louis, 2018), while another study found statements presented from the participant’s ingroup leader (*vs* from the outgroup) were perceived as less contradictory (Westen *et al.*, 2006). Perceived group membership and attitudes toward the ingroup or outgroup member also contribute to empathy-related behaviors towards the ingroup members (Hein *et al.*, 2010). This ingroup empathy bias is modulated in the anterior insula cortex, a region related to the impact of group membership on neural correlates of fear (Olsson *et al.*, 2005; Haaker *et al.*, 2016) and face processing (Golby *et al.*, 2001; Van Bavel *et al.*, 2008; Hein *et al.*, 2010). In contrast to empathy-related in-group bias, while watching a negatively evaluated outgroup member suffering pain, the activity of the anterior insula cortex (associated with empathy) has been found to be decreased, and activity in nucleus accumbens (NAcc) (associated with reward processing) was increased, suggesting that watching a negatively evaluated outgroup member receiving pain was processed in a reward-related manner (Hein *et al.*, 2010).

One perceptual and non-perceptual-based dimension in group perception that has been extensively investigated is social status (Karafin *et al.*, 2004; Cloutier *et al.*, 2008; Magee and Galinsky, 2008; Zaki *et al.*, 2011). Inference of status can be determined through observed demonstrations of skill, knowledge, generosity or prestige-related social competencies (e.g. affiliative tendency and morality (Mattan *et al.*, 2017) (see section social perceptual system regarding perceptual social status). Unlike for perceptual-based evaluations, status-based evaluations frequently engage regions known to support person evaluation (e.g. vmPFC) and reward/reinforcement learning (e.g. VS). Other regions involved in affective responses (e.g. amygdala and insula) and mentalizing (e.g. dMPFC, TPJ, STS/superior temporal gyrus (STG) and ATL) has also been associated with status conveyed through person-knowledge.

Other non-perceptual-based cues, such as personality traits, the knowledge of a person’s influence over others, their political opinions or their financial status also influence how group evaluations are formed. It has been suggested that the brain tracks discrepancies between a person’s behavior and the behavior that is expected based on their trait impressions (e.g. competence, trustworthiness and generosity: Boorman *et al.*, 2013; Hackel and Amodio, 2018; Morelli *et al.*, 2018). Several studies have revealed distinct ways in which the brain tracks the traits of others—one is associated with the conceptual representation of others and one tracks the value associated with individual’s traits. For example, one study found that—based on the positive or negative feedback received from another person in different contexts—the value of the person, as well as higher level trait inferences, is encoded in the VS (Mende-siedlecki *et al.*, 2013). However, the trait inferences additionally involve a broader network, including right temporoparietal junction (rTPJ), precuneus, inferior parietal lobule and ventrolateral PFC, regions previously identified as involved in more explicit forms of trait updating (Mende-siedlecki *et al.*, 2013). Overall, several networks seem to be involved in group perception involving perceptual, affective, cognitive systems and ToM (Eres and Molenberghs, 2013; Amodio, 2014).

### Integration of social computations

**Fig. 1.**
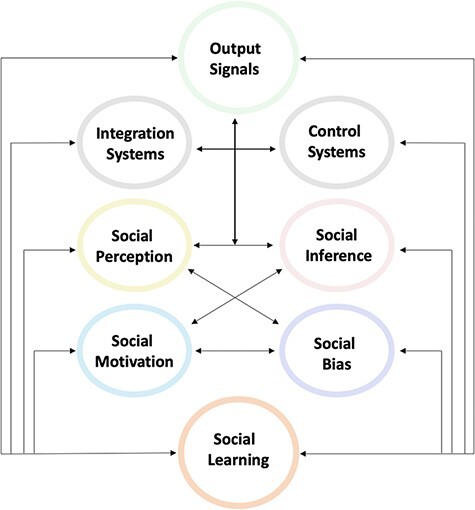
Multiple processes involved in social interaction. Perceptual signals and inferential processes are influenced by social drives and biases. These draw on learning systems that update and modify social behavior. Together, these processes are integrated to produce an output or social signal (e.g. facial expression and speech). These output systems are also modulated by control systems that filter social signals.

In reviewing the six computational aspects entailed by social interactions, we have seen some key themes emerge. First, processing depends upon distributed brain systems; particularly those involved in perspective-taking, social signals, and emotional and goal-directed behavior. These systems are exemplified by an engagement of face processing in fusiform areas, action observation in the extended mirror neuron system, subjective value signals in the medial PFC and the striatum, interoceptive inference in the anterior insular, and the extended reward system including subcortical systems, such as the amygdala. So, what principles could account for this plurality of brain systems—and what principles could be brought to bear on their functional integration? The goal of this section, therefore, is to explain the underling processes, as well as the integration, of perception and inferential system during social interaction (see Figure 1).

#### Active inference.

The account on offer here is based upon the notion of active inference; namely the view that all action and perception are in the service of minimizing uncertainty or maximizing model evidence (Friston *et al.*, 2011, 2017b). These complementary but equivalent perspectives inherit from a number of theories; in particular, the Bayesian brain hypothesis (Knill and Pouget, 2004) and the principle of maximum efficiency in information processing (Barlow, 1974; Optican and Richmond, 1987). The basic idea is that the brain actively constructs explanations for its sensory inputs, using a hierarchical generative model—that generates predictions of what would be sensed if the brain had correctly inferred states of affairs in the external world [Gregory, 1980; Kahl, R. (1971)]. There is a large literature on various neuronal process theories that underwrite this sort of inference, including predictive coding and belief propagation in cortical and subcortical hierarchies (Bastos *et al.*, 2012; Friston *et al.*, 2017a; Shipp, 2016). From our perspective, there are two key themes. First, the architecture of the brain recapitulates the architecture of the generative models used to predict sensory outcomes in all conceivable modalities over which it has control (Conant and Ross Ashby, 1970; Mansell, 2011). Second, if the social brain is associated with this kind of architecture, it must have some special properties. In other words, if the brain can predict all the consequences of social interactions, it means that the requisite generative model must be capable of generating predictions in the exteroceptive domain (for social inference based, for example, on facial expressions and nonverbal cues); it must be able to predict outcomes in the interoceptive domain (appropriate for inferences based upon affiliative touch and autonomic responses during prosocial engagements [Seth and Friston, 2016; Fotopoulou and Tsakiris, 2017)]. Finally, it clearly has to make predictions in the proprioceptive domain to enable motor acts, particularly, of communication, such as speech and nonverbal forms of exchange.

#### Active inference and the self.

In short, the special aspect of the social brain is that it has to accommodate every consequence of being a ‘self’. Indeed, the whole notion of minimal selfhood can be cast as a hypothesis used by the brain to explain for the myriad of sensory signals encountered during social exchange (Limanowski and Blankenburg, 2013; Seth and Critchley, 2013). Heuristically, what this means is that the brain infers that the self is the most probable cause of the exteroceptive, interoceptive and proprioceptive sensory signals to which it is privy. The picture that emerges here is of a deep hierarchical generative model that generates all modalities. A generative model is, technically, a probabilistic specification of how causes in the outside world generate sensory consequences (Hinton, 2007). Conversely, perceptual inference and synthesis corresponds to Bayesian model inversion, namely, inferring the causes from sensory consequences. Technically, this involves the maximization of the evidence for our models of the sensorium—that can be articulated as a minimization of variational free energy (i.e. a mathematical bound on model evidence) (Dayan *et al.*, 1995; Friston *et al.*, 2006). This can be thought of more simply as the minimization of surprise or prediction errors through neuronal message passing among different levels of cortical and subcortical hierarchies.

This view suggests that a generative model that starts with ‘me’ as the cause of my sensations will, when inverted, look as if I am assimilating and integrating multiple sensory modalities in the exteroceptive and interoceptive domains. If one also adds proprioception to this inference, I am effectively generating predictions about my own action, either in the autonomic or motor domain (Baker *et al.*, 2009; Friston *et al.*, 2011; Seth, 2014). This is referred to as active inference. When the perceptual synthesis implied by belief updating under such generative models includes interoceptive signals—as in affiliative and nurturing social interactions—we come to the notion of interoceptive inference (Barrett and Simmons, 2015; Fotopoulou and Tsakiris, 2017; Allen *et al.*, 2019). The term coined above—social inference—is meant to imply that the sort of active inference required for social exchange is of the broadest, multimodal nature conceivable, subsuming interoceptive inference and all other forms of inference in the service of modeling me and my interactions with you. On this view, the brain systems reviewed above start to make perfect sense—as heteroarchical subgraphs of a hierarchical graphical generative model, ultimately integrated under a supraordinate level of self-modeling. So, what does this say about how all the subsystems involved coordinate social perception, inference, communication and learning?

In brief, social perception rests upon exactly the same systems involved in nonsocial perception, but with a special emphasis on inferring the sensory cues supplied by ‘creatures like me’. Social influences, such as mentalizing, can—as the active inference story goes—be explained by repurposing generative models of my own behavior to explain yours; much in the sense of simulation theories and mirror neuron theories reviewed above. Put another way, communication and ToM become much easier if we have a shared narrative such that models of my behavior become models of your behavior—enabling ‘me’ to efficiently and accurately infer ‘our’ behavior (Friston and Frith, 2015). Clearly, to select the appropriate model of shared narratives means that I have to first infer that you are like me. This places the social perception, identity of agents and group membership center stage, in facilitating this particular aspect of social inference. That is, I first have to infer that you are like me before I can use my models of how I would behave to infer your intentions and state of mind. This high-level form of active inference comes along with some special considerations that we now consider in terms of social attention, joint attention and sensory attenuation.

#### Self-modeling and mental action.

Above, we considered the social brain as making inferences about states of affairs in a social econiche by maximizing the evidence for (or minimizing the variational free energy of) a hierarchical model of a world populated by ‘creatures like me’. Mathematically, this can be described as message passing on a graphical description of the generative model (i.e. a neural network), where this message passing corresponds to neuronal communication over extrinsic (between cortical area) connections and the intrinsic connectivity of canonical microcircuits (Bastos *et al.*, 2012; Shipp, 2016; Friston *et al.*, 2017b). In predictive coding formulations of this message passing, it is generally assumed that inference proceeds via reciprocal message passing between the levels of the hierarchical model. In particular, predictions are sent down from one level to the next that try to predict representations on the lower level. The resulting mismatch or prediction error is then returned to the higher level to induce belief updating or revisions of Bayesian beliefs encoded by neuronal activity (Shipp, 2016). This recurrent message passing/mediated by ascending streams of prediction errors and descending counter streams of predictions looks a lot like recurrent connectivity in cortical hierarchies in the brain (Hilgetag *et al.*, 2000).

So how is this message passing coordinated? In other words, how do we select those ascending signals that will update Bayesian belief representations in the right kind of way? Under active inference, the right kind of way corresponds to Bayes optimal inference, where the various sources of prediction errors and implicit information are weighted according to their reliability or precision (Knill and Pouget, 2004; Feldman and Friston, 2010; Parr and Friston, 2017). Physiologically, this corresponds to a delicate and fundamentally important control of postsynaptic gain or excitability of the neuronal populations broadcasting messages from one level to the next. Psychologically, this has been associated with attentional selection or attentional gain, and indeed, the complement, namely attenuation (such as in sensory attenuation) (Kok *et al.*, 2012; Brown *et al.*, 2013; Wiese, 2017). In short, the coordination of message passing in a hierarchical generative model rests upon context-sensitive predictions of the precision of various sources of information. In turn, this means that there must be a generative model of the precision or confidence afforded under different sorts of information.

This may sound obvious, but it has some profound implications for the nature of social inference. In brief, it means that we have the capacity to act upon our own hierarchical inference by selectively gating different sorts of information in a context-sensitive fashion. Many people consider this a form of mental action (Limanowski and Friston, 2018), much like the premotor theory of attention (Rizzolatti *et al.*, 1987). In short, mental action can be regarded as a covert action that samples the right kind of hierarchical information to make the best inferences about the (social) world based upon multisensory cues that are deconstructed in increasingly abstract and amodal levels. There are three reasons why this particular aspect of social inference has a special relevance for social cognition. First, forming representations about the precision or confidence ascribed to the contents of my representations is, effectively, a belief about beliefs and a formal sort of metacognition (Fleming *et al.*, 2012; Shea *et al.*, 2014). As such, it brings us close to a (possibly subpersonal) form of self-modeling that has an enactive—if covert—aspect. In fact, one could argue, that any (minimal) sense of self would be redundant unless it entailed a deployment of mental action and precision control over hierarchical processing (Limanowski and Blankenburg, 2013; Limanowski and Friston, 2018).

The second reason that this form of covert action is particularly important for the social brain is in communication and turn-taking (Wilson and Wilson, 2005; Ghazanfar and Takahashi, 2014). In brief, the ability to engage in verbal exchange, under a shared narrative, depends upon the alternating augmentation and attenuation of our sensory signals. This follows from the need to attenuate the sensed consequences of our own action—that would otherwise confound the fluent expression of motor reflexes (and indeed autonomic reflexes). Put simply, if I want to listen, I have to attenuate my proprioceptive predictions; otherwise I would find myself speaking (c.f., echolalia). Conversely, if I want to speak, I have to suspend that attenuation, while you are listening; see Friston and Frith (2015) for a simulation of this ‘turn-taking’. Furthermore, to use models of my own body to infer your intentions based upon what I see you doing, I have to attenuate the prediction errors that would ensue from proprioceptive predictions; otherwise I would overtly mirror your movements (i.e. echopraxia); see Friston *et al.* (2011) for a simulation of ‘action understanding’.

Active inference allows for a parsimonious explanation of many human behavioral tendencies noted above, especially prosocial behavior and motivation. For example, humans tend to be motivated to cooperate with conspecifics, especially with members of their ingroup, and to dislike those from outgroups. In human social groups, an especially important prior belief is that other human agents in our ingroup will align their mental states with our own and vice versa. This has been proposed as one of the prior beliefs that define the human cooperative phenotype and that make communication possible (Vasil *et al.*, 2019). Human cooperation and distinctly human forms of cooperative communication, then, are underwritten by the shared belief—formalized in active inference and harnessed in the generative models that are species-typical of humans—that ‘we are the same kind of creature, inhabiting the same cultural niche’ and that therefore ‘we should align with one another’.

There are many other fascinating issues that attend the augmentation and attenuation of precision (i.e. attention) in this setting, specifically, the notion of joint attention in higher-order forms of social inference (Moll and Meltzoff, 2011). However, we will conclude this subsection by noting a particularly important aspect of precision control, namely, its intimate relationship to emotional inference and interoception.

In brief, much of social interaction has a substantial interoceptive component, hence the frequent reference to the anterior insular (Paulus and Stein, 2006; Craig, 2013; Gu *et al.*, 2013; Seth and Friston, 2016; Fotopoulou and Tsakiris, 2017). It may be that our sense of self and feelings (induced by another) are inferences that provide the best explanation for the myriad of autonomic signals inherent in any prosocial exchange (Barrett and Simmons, 2015; Fotopoulou and Tsakiris, 2017). These feeling states both inform and are informed by various levels of confidence or uncertainty about what will happen next or what one should do next. This takes us in the direction of emotional inference and the psychopathology of stress (and avoidance)—all of which are especially relevant for social inference and learning (Peters *et al.*, 2017). However, we will now close with a slightly broader perspective that takes us beyond the brain (and body) but still pursues the overall goal of inference and the minimization of uncertainty.

#### The social brain and cultural niche construction.

In recent years, there has been a move toward generalizing the principles of active inference beyond the brain, to cover things like variational ethology, niche construction and deontic value (Bruineberg and Rietveld, 2014; Constant *et al.*, 2018, 2019; Badcock *et al.*, 2019; Veissière *et al.*, 2019). This extension nicely subsumes some of the more encultured aspects of social learning and inference reviewed above. The basic idea here is that if one reduces (social) cognition to the minimization of uncertainty (or the maximization of expected model evidence), a simple explanation for much of ethology and the nongenetic inheritance described above starts to emerge.

In brief, if we associate model evidence with adaptive fitness, then natural selection just becomes Bayesian model selection (Frank, 2012). On this view, natural selection is driven by the imperative for self-evidencing (Hohwy, 2016), namely, making the world as predictable and as learnable as possible. We have seen beautiful examples of this above, in terms of mimicry and other forms of socially mediated econiche construction. There is a formal treatment of this form of cultural niche construction under active inference that unfolds at two levels. The first is in a reciprocal exchange between a phenotype and her environment such that as an agent learns about her world, the world ‘learns’ about the phenotype to which it plays host, in the sense that it comes to mirror the statistical structure of the actions of its denizens by accumulating traces of those actions. A compelling example of this is the phenomena of desire paths or elephant paths: these correspond to paths (e.g. across a field or park) that are worn down by frequent use. The emergence of desire paths could be seen in terms of niche construction, in the sense that they reflect the enacted desires and predicted (locomotive) behavior of phenotypes. On the other hand, they also provide ‘deontic’ cues that encourage walking and the very emergence and maintenance of these paths in and of themselves (Constant *et al.*, 2018), where ‘deontic’ cues are cues endowed with a shared value for a given community and which have an obligatory or deontic character. For example, humans learn to stop at red traffic lights, which function as a deontic cue that conveys the value of a given policy (in this case, stopping at a red light) for all enculturated members of the community. In short, the environment is effectively remembering the sort of behavior which adaptive phenotypes exhibit. The implicit circular causality can now be extended to interpersonal exchange and a similar ‘offloading’ of the sorts of phenotypes found in this niche—that can be lifted to the level of semiotics (e.g. traffic lights and signs in our lived environments) (Constant *et al.*, 2019) and, ultimately, social exchange (Shea *et al.*, 2014; Veissière *et al.*, 2019). The underlying message here is that the social brain may be a product of hierarchical inference—not just within the skull—but in the context of coevolution with conspecifics and a shared environmental niche. At its heart, all of the processes entailed by cultural niche construction and ‘group living’ are quintessentially social.

## Concluding remarks

A clear goal of neuroscience and artificial intelligence is to understand how the brain functions during social interactions. By dissecting the social brain into its core components and rebuilding it to examine how these components work together, we can begin to understand how the human brain computes input and output signals to form coherent social behaviors. A future goal of social neuroscience is to provide better psychological, computational and anatomical models of the social brain in action, a goal that will involve innovations in paradigm and technical development. A great start is to build paradigms that reflect real social interaction or more immersive social environments and use techniques that provide better temporal and spatial resolution.
